# Dryland Crop Classification Combining Multitype Features and Multitemporal Quad-Polarimetric RADARSAT-2 Imagery in Hebei Plain, China

**DOI:** 10.3390/s21020332

**Published:** 2021-01-06

**Authors:** Di Wang, Chang-An Liu, Yan Zeng, Tian Tian, Zheng Sun

**Affiliations:** 1 Institute of Agricultural Resources and Regional Planning, Chinese Academy of Agricultural Sciences, Beijing 100081, China; wangdi02@caas.cn (D.W.); iszengyan@163.com (Y.Z.); tiant0025@163.com (T.T.); 2 Key Laboratory of Agricultural Remote Sensing, Ministry of Agriculture and Rural Affairs, Beijing 100081, China; 3 State Geospatial Information Center, Beijing 100070, China; liuchangan@caas.cn

**Keywords:** dryland crop classification, PolSAR, multitype feature, RADARSAT-2, polarization decomposition, Hebei plain

## Abstract

The accuracy of dryland crop classification using satellite-based synthetic aperture radar (SAR) data is often unsatisfactory owing to the similar dielectric properties that exist between the crops and their surroundings. The main objective of this study was to improve the accuracy of dryland crop (maize and cotton) classification by combining multitype features and multitemporal polarimetric SAR (PolSAR) images in Hebei plain, China. Three quad-polarimetric RADARSAT-2 scenes were acquired between July and September 2018, from which 117 features were extracted using the Cloude–Pottier, Freeman–Durden, Yamaguchi, and multiple-component polarization decomposition methods, together with two polarization matrices (i.e., the coherency matrix and the covariance matrix). Random forest (RF) and support vector machine (SVM) algorithms were used for classification of dryland crops and other land-cover types in this study. The accuracy of dryland crop classification using various single features and their combinations was compared for different imagery acquisition dates, and the performance of the two algorithms was evaluated quantitatively. The importance of all investigated features was assessed using the RF algorithm to optimize the features used and the imagery acquisition date for dryland crop classification. Results showed that the accuracy of dryland crop classification increases with evolution of the phenological period. In comparison with SVM, the RF algorithm showed better performance for dryland crop classification when using full polarimetric RADARSAT-2 data. Dryland crop classification accuracy was not improved substantially when using only backscattering intensity features or polarization decomposition parameters extracted from a single-date image. Satisfactory classification accuracy was achieved using 11 optimized features (derived from the Cloude–Pottier decomposition and the coherency matrix) from 2 RADARSAT-2 images (acquisition dates corresponding to the middle and late stages of dryland crop growth). This study provides an important reference for timely and accurate classification of dryland crop in Hebei plain, China.

## 1. Introduction

A regional classification map of crops type provides an important basis for estimating the areal coverage, monitoring the growth, and forecasting various crop yields. Timely availability of accurate information regarding the spatial distribution of crop types plays a very important role in scheduling agricultural management policies, ensuring national food security, and evaluating ecological function [1,2]. Satellite-based remote-sensing techniques have proven very effective for mapping crops owing to their advantages of wide spatial coverage, periodic observation, high efficiency, and low cost [3,4,5]. Optical imagery such as that acquired by the Landsat-8, Sentinel-2, and Gaofen-1 satellites has become the main data source for classifying crop types and determining their spatial distribution [6].

The Hebei plain, covering an area of 81,600 km^2^, is an important grain and cotton-producing region in China [7]. Cultivated land is a predominant land use type and dryland crops, such as maize and cotton, have been widely planted in Hebei plain for a long time. Affected by the monsoon climate of medium latitudes, 80% of average annual precipitation falls in this region during July–September, which just covers the main growing periods of maize and cotton [8]. Unfortunately, the availability and quality of optical remotely sensed imagery covering this region are often very poor, owing to the cloudy/rainy weather that occur frequently during the critical phenological stages of this two typical dryland crops in Hebei plain [9]. Consequently, optical remote-sensing techniques are inadequate for accurate and timely monitoring of these crops.

As an active microwave remote-sensing technology, the synthetic aperture radar (SAR) has 24-h all-weather observation capability, making it a promising tool for crop monitoring in regions that experience frequent cloud cover. Restricted by sensor techniques, early studies that used SAR remote sensing to extract crop-related information involved single-polarization (e.g., HH or VV (horizontal or vertical co-polarization)) data that were commonly acquired by satellite-based orbital radar systems, e.g., ERS-1 (the first European Remote Sensing Satellite), ERS-2 (the second European Remote Sensing Satellite), JERS-1 (Japanese Earth Resources Satellite 1), and RADARSAT-1 [1]. As an important feature, the backscattering coefficient (σ^0^) was often used to identify crops during this period of research [10,11,12]. In particular, considerable attention focused on identification of rice using single-polarized SAR data [13,14,15]. This was because the σ^0^ of rice varies substantially throughout the growing season, and because the dielectric properties of a paddy field are markedly different from those of other crop types. However, little attention has been given to dryland crop classification using SAR imagery because of the complex planting structures and similar dielectric properties of such crops.

Distinguishing crops in SAR imagery is dependent primarily on their geometric configuration (e.g., size, shape, and leaf inclination), the cropping system (e.g., planting density and row direction), and the dielectric properties of the crop canopy and the underlying background soil [16]. However, a single- or dual-polarization SAR system has no capability to provide sufficient information with which to characterize such crop-related differences. Consequently, the accuracy of crop classification using single-polarized SAR data remains low, especially for dryland crops. In comparison with the single- or dual-polarization approaches, polarimetric SAR (PolSAR, also called quad-polarimetric or fully polarized SAR) with four polarization channels can provide richer information on the scattering mechanisms of various targets with different shapes and structures that can be used to distinguish them. Therefore, this approach has been used widely during the previous 10 years to improve crop classification accuracy [17,18,19,20,21,22].

Polarimetric target decomposition methods are used commonly to extract various polarization features of land surface objects from PolSAR imagery. Depending on whether there is a change in the scattering properties of a target, polarimetric decomposition methods can be classified into two main categories: coherent decomposition that is based on a single-look Sinclair scattering matrix, and incoherent decomposition that is based on a multilook scattering matrix, i.e., the coherency matrix or the covariance matrix [23,24]. Usually, coherent decomposition is applied to analyze a “pure single target” that has deterministic or stationary scattering characteristics [24]. Representative approaches include the Pauli, Krogager, and Cameron decompositions. In contrast, incoherent decomposition is applied to investigate distributed targets with probabilistic scattering properties. Accordingly, representative approaches include eigen-based decomposition (e.g., Cloude–Pottier (C–P) decomposition) and model-based decomposition (e.g., Freeman–Durden (F–D) and Yamaguchi decomposition). As crops growing on farmland represent distributed targets, their polarization features are usually extracted using an incoherent decomposition approach. Many previous studies using PolSAR data have demonstrated that crop classification accuracy can be improved using various features derived from polarimetric decomposition [25,26,27]. For example, using L-band PolSAR data, McNairn et al. [25] found that the polarization features derived using the C–P and F–D decomposition methods produced superior accuracy in classification of crops (e.g., corn, soybeans, cereals, and hay-pasture) relative to that achieved using linear polarization. Many features can be extracted from PolSAR imagery using polarimetric target decomposition methods; however, it remains unclear which features are most suitable for dryland crop classification.

Generally, most crops have a growing season of several months. In this short time, different crops show distinct variations in terms of their geometric shape, structure, and dielectric properties with evolution of the phenological period [28]. Therefore, multitemporal SAR imagery has often been employed to improve crop discrimination capability and classification results. Huang et al. [21] used seven polarimetric RADARSAT-2 images of southwestern Ontario (Canada) to analyze the scattering mechanisms of six land-cover types that included wheat, soybean, and corn. They acquired a classification map with overall accuracy of 87.5% based on the distinct scattering mechanisms of different land cover types. To improve classification accuracy, Xie et al. [24] acquired a time series of 11 polarimetric RADARSAT-2 SAR images of an agricultural site in London, Ontario (Canada), and they employed the Neumann decomposition approach to extract the discriminant features and the random forest (RF) method to classify 9 land-cover types. The overall accuracy (OA) and the Kappa coefficient of the classification of the nine land-cover types in their study were 94.12% and 0.92, respectively. In comparison with single-date data, better classification accuracy can be achieved when using multitemporal SAR images [29,30,31]. However, any improvement in accuracy is often accompanied by high costs related to imagery acquisition and increased workload associated with data processing owing to the increase of the image time phase. Consequently, little attention has been paid to the time phase selection or optimization of SAR imagery for use in crop classification, especially dryland crops.

The classifier is an important component in any image classification procedure. After discriminant features have been determined, an appropriate classification algorithm is required to produce an accurate crop map. The various approaches that have been developed for crop classification using PolSAR imagery can be divided broadly into two categories: (1) algorithms based on statistical models and (2) algorithms based on machine learning theory. Conventional statistical algorithms include the maximum likelihood classifier [32], Wishart classifier [22,33], and Hoekman and Vissers classifier [34]. Of these, the Wishart classifier is adopted most commonly for crop classification using the PolSAR data. However, statistical methods such as these depend on the assumption that the pixels to be classified have a specific probability distribution, which hinders the performance of the algorithm. Machine-learning algorithms do not require determination of the probability distribution function of the pixels in the imagery and hence overcome the shortcomings of statistical algorithms [16]. In comparison with statistical methods, machine-learning methods have been proven to produce higher classification accuracy [16,35,36,37,38,39]. Owing to their high accuracy and ease of operation, the support vector machine (SVM) and RF approaches are two of the machine learning methods used most widely for crop classification. However, little consideration has been given to the determination of which method is most suitable for dryland crop classification using satellite-based PolSAR imagery.

Based on RADARSAT-2 PolSAR imagery, this study compared the accuracy of classification of typical dryland crops (i.e., maize and cotton) at different phenological stages in Jizhou county, Hebei plain, China. The importance of various features extracted from the multitemporal PolSAR data for crop discrimination was investigated, and the optimal observation periods, features, and algorithms for dryland crop classification using PolSAR data were established. The remainder of this paper is organized as follows. Section 2 introduces the study site and the data used. Section 3 reports on the various features considered and their extraction methods, as well as the classification algorithms. Section 4 presents analysis of the experimental results. Finally, a discussion and our conclusions are provided in Section 5 and Section 6, respectively.

## 2. Study Site and Dataset

### 2.1. Study Site Description

**Figure 1:**
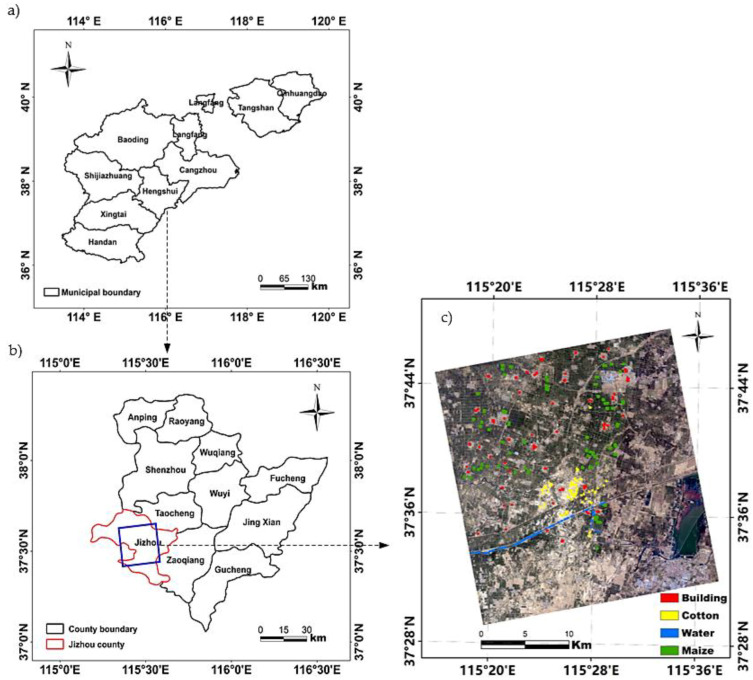
(**a**) Hebei plain, (**b**) location of the study area and (**c**) the distribution of sample plots shown on a GF-1 PMS image.

The study site (37°30′–37°45′ N, 115°15′–115°35′ E; Figure 1) is located in Jizhou county, Hebei province, China. This county was chosen as the study area because it lies in the middle of Hebei plain and is representativeness of this plain in crop planting characteristics. Jizhou county has a total land area of 918 km^2^, and its terrain is flat and open with an elevation range of approximate 23–29 m. Sandy loam and light loam comprise the main soil types within the study area, which are very suitable for the cultivation of dryland crops. The region has a semi-humid continental monsoon climate. The average annual temperature is 13.10 °C. The average annual precipitation is approximately 461.80 mm, most of which falls during July–September.

**Figure 2:**
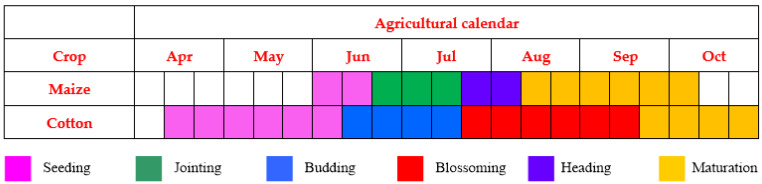
Phenological stages of two dryland crops (maize and cotton) in the study area.

Cropland, which is the main land-use type, accounts for approximately 65% of total land area of the study site. Winter wheat, summer maize, and cotton are the three main crops cultivated on the cropland. After harvesting of the winter wheat, summer maize is sown in the same plots. We selected maize and cotton as typical dryland crops with which to conduct a classification using PolSAR data because their growing period is primarily in the rainy season. The specific phenological periods of maize and cotton are shown in Figure 2. In addition to cropland, built-up areas and water bodies comprise the two other main land-cover types in the study area.

### 2.2. RADARSAT-2 Data Description

RADARSAT-2 full polarimetric SAR data were chosen for the dryland crop classification because of their high spatial resolution and full polarization modes (i.e., HH, HV (horizontal transmission and vertical reception), VH (vertical transmission and horizontal reception), and VV). Launched in December 2007, RADARSAT-2 has a 24-d revisit interval. A sequence of RADARSAT-2 scenes of the study area was acquired in 2018. Images acquired on the following three dates were selected for analysis: 14 July, 7 August, and 24 September 2018, because these dates represent key phenological stages of maize and cotton. All scenes were acquired in Fine Quad-Pol mode and as Single-Look-Complex products with spatial resolution of 5.2 m in range and 7.6 m in azimuth. In all cases, the incidence angle of the images was 26.6° and their swath range was 25 km × 25 km. Further details regarding the three scenes and the corresponding phenological stages of the two studied dryland crops are shown in Table 1.

### 2.3. Ground-Truth Data Description

Field surveys of the study area were undertaken three times during July–September 2018, and the date of each survey was the same as that of the acquisition of the RADARSAT-2 scenes. Each survey collected information on crop type, leaf area, and plant height (PH). Sample plots were selected according to their representativeness of the four typical land-cover types (i.e., maize, cotton, water, and buildings) and the spatial distribution of the various land cover types. A single land-cover type was required in each sample plot. Moreover, for the mirror land cover type, such as cotton, the size of sampled field plots should not too small to cover enough pixels. Overall, 418 sample plots distributed randomly throughout the entire study area were chosen; these comprised 101 maize sample plots, 115 cotton sample plots, 100 water sample plots, and 102 built-up area sample plots (Figure 1). The locations of all sample plots were recorded using a handheld differential global positioning system (Trimble TDC 100, Trimble Inc., Sunnyvale, CA, USA) with a positioning accuracy of 1 m, and then their boundaries were delineated and further digitized using a GF-1 PMS (Panchromatic and Multispectral) image with 2-m spatial resolution. All sample plots were divided into two parts: 66.7% of the samples were chosen randomly as training points, and the remaining samples were used for validation. In addition to establishing land-cover type, the leaf area and plant height of crop samples were also measured using a LAI-2000 plant canopy analyzer (LI-COR, Inc., Lincoln, ND, USA) and steel tape, respectively, to analyze the changes of crop geometrical structure with phenological evolution. Three maize or cotton plants in each crop sample plot were chosen at random for the measurements of leaf area and PH, and the averages of the values obtained from the three plants were considered representative of one crop sample plot. A detailed description of the field survey data is presented in Table 2.

## 3. Methodology

**Figure 3:**
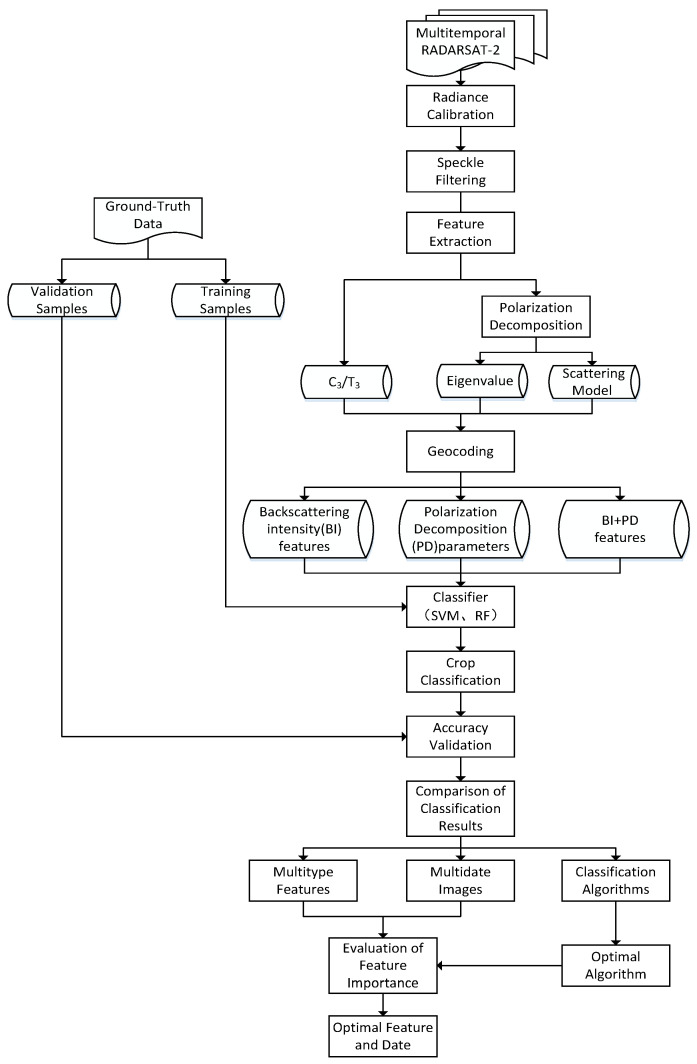
Workflow of the dryland crop classification methodology.

The workflow of the dryland crop classification scheme comprised five principal steps (Figure 3). (1) Image preprocessing, which included radiance calibration, filtering, geocoding, and registration. (2) Feature extraction in which various features were extracted using polarization decomposition methods based on the coherency matrix or the covariance matrix. (3) Classification and validation, whereby land-cover classification was conducted using different features and classifiers at different phenological stages of the crops, following which the classification accuracy was tested using the validation samples. (4) Comparison and evaluation in which the accuracy of the dryland crop classification using different times, features, and classification algorithms were compared and evaluated. (5) Optimization of the dryland crop classification scheme. The importance of various features was assessed quantitatively using the RF model, and then the optimal features, time, and classifier were identified based on the results obtained in step (4).

### 3.1. Image Preprocessing

The SAR image preprocessing comprised the following steps: (1) radiance calibration, (2) speckle filtering, (3) geocoding, and (4) image registration. The refined Lee filter with a 7 × 7 window was used for speckle reduction. The range-Doppler approach and Shuttle Radar Topography Mission digital elevation model data with 30-m spatial resolution were used for SAR image geocoding. After resampling, the spatial resolution of the SAR image was set as 5 m × 5 m and the projection coordinate system as WGS (World Geodetic System) 84 UTM-Zone 50. Registration was performed for the RADARSAT-2 polarimetric images obtained at the three acquisition times using 60 control points and the GF-1 PMS image mentioned in Section 2.3. The radiance calibration and speckle filtering steps of the image preprocessing procedure were conducted using PolSARpro 5.1.2 software (University of Rennes 1, Rennes, France). The SAR images were geocoded using NEST 4C software (Array Systems Computing Inc., Los Angeles, CA, USA), and image registration was executed using ENVI 5.3 software.

### 3.2. Feature Extraction with RADARSAT-2 Polarimetric Imagery

In comparison with single- or dual-polarization SAR imagery, additional features can be extracted when using PolSAR data. In this study, three feature types were extracted from the RADARSAT-2 PolSAR imagery: (1) features derived using the coherency matrix and the covariance matrix, (2) features extracted using the polarization target decomposition approach based on eigenvectors, and (3) features extracted using the polarization target decomposition approach based on the scattering model.

#### 3.2.1. Feature Extraction Using the Coherency Matrix and Covariance Matrix

The energy information received by the SAR system reflects the scattering characteristics of ground objects. Moreover, the echo intensity of various ground objects is also different. The backscattering coefficient (σ^0^) is a classical index for describing the echo intensity of ground objects. For distributed targets such as crops, the coherency matrix (*T*_3_) and the covariance matrix (*C*_3_) are used widely in analyses of the scattering mechanism. Moreover, the main diagonal elements of the two matrices also represent the energy information of various backscattering types and polarization modes. In *T*_3_, the main diagonal element comprising *T*_11_, *T*_22_, and *T*_33_ denotes the energy value of surface, double, and multiple scattering, respectively. Similarly, in *C*_3_, the main diagonal element comprising *C*_11_, *C*_22_, and *C*_33_ denotes the backscattering coefficient at HH, HV/VH, and VV polarization mode, respectively. Therefore, we selected the six main diagonal elements as features for dryland crop classification because they reflect the scattering characteristics of ground objects. The coherency matrix (*T*_3_) and covariance matrix (*C*_3_) can be expressed as follows [40,41]:

(1) $$T_{3}=\frac{1}{2}\left[\begin{array}{ccc} \langle {\left|S_{\mathrm{HH}}+S_{\mathrm{VV}}\right|}^{2}\rangle & \langle (S_{\mathrm{HH}}+S_{\mathrm{VV}}){\left(S_{\mathrm{HH}}-S_{\mathrm{VV}}\right)}^{*}\rangle & 2\langle \left(S_{\mathrm{HH}}+S_{\mathrm{VV}}\right)S_{\mathrm{HV}}^{*}\rangle \\ \langle \left(S_{\mathrm{HH}}-S_{\mathrm{VV}}\right){\left(S_{\mathrm{HH}}+S_{\mathrm{VV}}\right)}^{*}\rangle & \langle {\left|S_{\mathrm{HH}}-S_{\mathrm{VV}}\right|}^{2}\rangle & 2\langle \left(S_{\mathrm{HH}}-S_{\mathrm{VV}}\right)S_{\mathrm{HV}}^{*}\rangle \\ 2\langle S_{\mathrm{HV}}{\left(S_{\mathrm{HH}}+S_{\mathrm{VV}}\right)}^{*}\rangle & 2\langle S_{\mathrm{HV}}{\left(S_{\mathrm{HH}}-S_{\mathrm{VV}}\right)}^{*}\rangle & 4\langle {\left|S_{\mathrm{HV}}\right|}^{2}\rangle \end{array}\right]$$

(2) $$C_{3}=\left[\begin{array}{ccc} \langle {\left|S_{\mathrm{HH}}\right|}^{2}\rangle & \sqrt{2}\langle S_{\mathrm{HH}}S_{\mathrm{HV}}^{*}\rangle & \langle S_{\mathrm{HH}}S_{\mathrm{VV}}^{*}\rangle \\ \sqrt{2}\langle S_{\mathrm{HV}}S_{\mathrm{HH}}^{*}\rangle & 2\langle {\left|S_{\mathrm{HV}}\right|}^{2}\rangle & \sqrt{2}\langle S_{\mathrm{HV}}S_{\mathrm{VV}}^{*}\rangle \\ \langle S_{\mathrm{VV}}S_{\mathrm{HH}}^{*}\rangle & \sqrt{2}\langle S_{\mathrm{VV}}S_{\mathrm{HV}}^{*}\rangle & \langle {\left|S_{\mathrm{VV}}\right|}^{2}\rangle \end{array}\right]$$

where 〈 〉 denotes the average value of the spatial or temporal set; | | is the determinant of a square matrix; * denotes the adjoint matrix of the matrices; and *S*_HH_, *S*_HV_, and *S*_VV_ are the values of the three elements located in the scattering matrix *S*, where *S* can be expressed as [41]:

(3) $$S=\left[\begin{array}{cc} S_{\mathrm{HH}} & S_{\mathrm{HV}}\\ S_{\mathrm{VH}} & S_{\mathrm{VV}} \end{array}\right]$$

#### 3.2.2. Feature Extraction Using the Cloude–Pottier Decomposition Method

The C–P decomposition method is the one of most widely used polarimetric decomposition approaches in which the coherency matrix (*T*_3_) is decomposed into three eigenvalues and their corresponding eigenvectors. Because various eigenvectors and eigenvalues represent different scattering types and their corresponding amplitudes, moreover, plenty of polarization decomposition parameters can be derived by the combinations of these eigenvalues and eigenvectors, therefore, we chose the C–P decomposition method to extract the polarization features for dryland crop classification. According to eigen decomposition theory [41], *T*_3_ can be expressed as follows:

(4) $$\left[T_{3}\right]=\overset{3}{\underset{i=1}{\sum }}\lambda_{i}u_{i}\cdot u_{i}^{*T}{}_{}^{}$$

where $\lambda_{i}$ and $u_{i}$ (*i* = 1, 2, 3) denote the eigenvalue and eigenvector, respectively, derived from the coherency matrix using the C–P decomposition approach. In the C–P decomposition process, each eigenvector $u_{i}$ can be expressed using four real angular parameters, as expressed in Equation (5) [41]:

(5) $$u_{i}={\left[\begin{array}{ccc} \cos\alpha_{i} & \sin\alpha_{i}\cos\beta_{i}e^{j\delta_{i}} & \sin\alpha_{i}\cos\beta_{i}e^{j\gamma_{i}} \end{array}\right]}^{T}$$

where α, β, δ, and γ are the four angular parameters. Here, $\bar{\alpha }$ denotes the average scattering angle, and $\bar{\beta },\;\bar{\delta },\;$ and $\bar{\gamma }$ define the polarization direction angle [42], which can be calculated as follows [42]:

(6) $$\left(\bar{\alpha },\bar{\beta },\bar{\delta },\bar{\gamma }\right)=\overset{3}{\underset{i=1}{\sum }}p_{i}\left(\alpha_{i},\beta_{i},\delta_{i},\gamma_{i}\right)$$

(7) $$p_{i}=\lambda_{i}/\overset{3}{\underset{i=1}{\sum }}\lambda_{i}\;$$

where $p_{i}$ is the probability of the eigenvalue $\lambda_{i}$. The eigenvalue and eigenvector are the main parameters extracted using the C–P decomposition method. In addition to the 10 aforementioned parameters, we again used the C–P decomposition approach to extract an additional 11 secondary parameters from T_3_ to analyze the scattering characteristics of various land cover types in the study area. The 11 secondary parameters were entropy (*H*), anisotropy (*A*), average eigenvalue ($\bar{\lambda }$), single-bounce eigenvalue relative difference (SERD), double-bounce eigenvalue relative difference (DERD), target randomness (*P*_R_), radar vegetation index (RVI), pedestal height (PH), Shannon entropy (SE), polarization component of SE (SE_p_), and intensity component of SE (SE_I_). These parameters were calculated using Equations (8)–(21) [41], and they can be acquired directly using the PolSARpro 5.1.2 software:

(8) $$H=-\overset{3}{\underset{i=1}{\sum }}p_{i}\log_{3}p_{i}$$

(9) $$A=\frac{\lambda_{2}-\lambda_{3}}{\lambda_{2}+\lambda_{3}}$$

(10) $$\bar{\lambda }=\frac{1}{3}\overset{3}{\underset{i=1}{\sum }}\lambda_{i}$$

(11) $$\mathrm{SERD}=\frac{\lambda_{S}-\lambda_{3_{\mathrm{NOS}}}}{\lambda_{S}+\lambda_{3_{\mathrm{NOS}}}}$$

(12) $$\mathrm{DERD}=\frac{\lambda_{D}-\lambda_{3_{\mathrm{NOS}}}}{\lambda_{D}+\lambda_{3_{\mathrm{NOS}}}}$$

(13) $$\lambda_{1_{\mathrm{NOS}}}=\frac{1}{2}\left\{\langle {\left|S_{\mathrm{HH}}\right|}^{2}\rangle +\langle {\left|S_{\mathrm{VV}}\right|}^{2}\rangle +\sqrt[{}]{{\langle |S_{\mathrm{HH}}|}^{2}\rangle -\langle {\left|S_{\mathrm{VV}}\right|}^{2}\rangle +4\langle \langle S_{\mathrm{HH}}S_{\mathrm{VV}}^{*}\rangle^{2}\rangle }\right\}$$

(14) $$\lambda_{2_{\mathrm{NOS}}}=\frac{1}{2}\left\{\langle {\left|S_{\mathrm{HH}}\right|}^{2}\rangle +\langle {\left|S_{\mathrm{VV}}\right|}^{2}\rangle -\sqrt[{}]{\langle {\left|S_{\mathrm{HH}}\right|}^{2}\rangle -\langle {\left|S_{\mathrm{VV}}\right|}^{2}\rangle +4\langle \langle S_{\mathrm{HH}}S_{\mathrm{VV}}^{*}\rangle^{2}\rangle }\right\}$$

(15) $$\lambda_{3_{\mathrm{NOS}}}=2\langle {\left|S_{\mathrm{HV}}\right|}^{2}\rangle$$

(16) $$P_{R}=\sqrt[{}]{\frac{3}{2}}\sqrt[{}]{\frac{\lambda_{2}^{2}+\lambda_{3}^{2}}{\lambda_{1}^{2}+\lambda_{2}^{2}+\lambda_{3}^{2}}}$$

(17) $$\mathrm{RVI}=\frac{4\lambda_{3}}{\lambda_{1}+\lambda_{2}+\lambda_{3}},\;0\leq \mathrm{RVI}\leq \frac{4}{3}$$

(18) $$\mathrm{PH}=\frac{min\left(\lambda_{1},\lambda_{2},\lambda_{3}\right)}{max\left(\lambda_{1},\lambda_{2},\lambda_{3}\right)},\lambda_{1}\geq \lambda_{2}\geq \lambda_{3},0\leq \mathrm{PH}\leq 1$$

(19) $$\mathrm{SE}\;=\log\left(\pi^{3}e^{3}\left|T_{3}\right|\right)\;={\mathrm{SE}}_{p}+{\mathrm{SE}}_{I}$$

(20) $${\mathrm{SE}}_{p}=3\log\left(\frac{\pi eI_{T}}{3}\right)=3\log\left(\frac{\pi e\mathrm{Tr}\left(T_{3}\right)}{3}\right)\;$$

(21) $${\mathrm{SE}}_{I}\;=\log\left(1-P_{T}^{2}\right)=\log\left(27\frac{\left|T_{3}\right|}{\mathrm{Tr}{\left|T_{3}\right|}^{3}}\right)\;$$

#### 3.2.3. Feature Extraction Using Scattering Model Decomposition Methods

As two typical scattering model decomposition methods, F–D and Yamaguchi decomposition have been used widely for polarization target decomposition. For F–D decomposition, a model is first constructed for three types of scattering mechanism (odd, double, and volume scattering) based on the coherency matrix (*T*_3_) or covariance matrix (*C*_3_), and then the contribution of the three scattering types to the total scattering power is estimated using this model. Three parameters were extracted using F–D decomposition in this study: odd scattering (Freeman-odd), double scattering (Freeman-dbl), and volume scattering (Freeman-vol). In comparison with F–D decomposition, Yamaguchi decomposition adds an additional helical scattering component. Therefore, four parameters were extracted when using the Yamaguchi decomposition approach. The multiple-component scattering model (MCSM) proposed by Zhang [42,43,44] is another decomposition approach in which a wire scattering component is added based on the Yamaguchi decomposition method; therefore, five parameters were extracted when using MCSM decomposition in this study. All parameters extracted by the above scattering model decomposition approaches can be derived using the PolSARpro 5.1.2 software. Overall, 39 features were derived from the RADARSAT-2 PolSAR data and their serial numbers are listed in Table 3.

### 3.3. Classification Algorithm

In this study, RF and SVM algorithms were used for the classification of dryland crops and other land-cover types because both algorithms are used widely in crop classification applications based on remotely sensed imagery.

The RF algorithm is an ensemble of many decision trees, and each decision tree is an elementary entity of this algorithm as well as a classifier. The classification result derived from each decision tree is first voted on, and then the class receiving the most votes is determined as the final classification result. The RF algorithm has many advantages in crop classification applications that include high classification accuracy, short running time, efficient handling of large numbers of input variables, resistance to overfitting, and fewer parameter settings. Two critical parameters must be set when performing RF classifications using EnMAP Box software: the number of decision trees and the number of features used for detecting each split during training of the decision trees. In this study, these values were set as 100 decision trees and the square root of the total number of input features, respectively. In addition, another advantage of the RF algorithm is assessment of the importance of all input variables, which is based on the value of the mean decrease in accuracy (i.e., the difference in prediction accuracy before and after permutation of the variable of interest) [7]. In this study, we also evaluated quantitatively the importance of various features extracted from the RADARSAT-2 PolSAR image for dryland crop classification using the RF algorithm.

The SVM approach is another machine-learning algorithm based on statistical learning theory. The main principle of this algorithm is determination of an optimal hyperplane for efficient discrimination of various classes of objects using training sample sets [45]. Similar to the RF algorithm, the SVM algorithm also has many merits in crop classification applications. For example, it is able to overcome certain problems such as a small number of samples, linear inseparability, and high-dimensional data. Moreover, high classification accuracy is often acquired when using this algorithm. Generally, several parameters (e.g., the kernel function and penalty factor) must be set when conducting SVM classification. However, we used a new image classification framework that is incorporated in the ENVI 5.5 software, and the optimal parameters were acquired by the training sample data; therefore, it was not necessary for the aforementioned parameters to be set in advance for the SVM classification. After performing the classification of the dryland crops and the other land-cover types using the RF and SVM algorithms, the classification accuracies and running times of the two algorithms were compared and assessed.

## 4. Results

### 4.1. Classification Performance with Only the Feature of Backscattering Intensity

The various accuracies and Kappa coefficients of the classification of the two dryland crops and other land-cover types when using six features (extracted from the coherency matrix and the covariance matrix) and two algorithms (RF and SVM) on three dates are listed in Table 4. In terms of the classification accuracy, the overall accuracy (OA) and Kappa coefficient of each of the four typical ground objects are not high on the three different dates, i.e., they vary in the ranges of 77.31–81.20% and 0.619–0.687%, respectively, whichever algorithm was used for the classification. This indicates that satisfactory accuracy could not be achieved when using only backscattering intensity features. For the four land-cover types, water has the highest classification accuracy, followed in descending order by maize, buildings, and cotton. Owing to its smooth surface, the backscattering intensity of water is markedly lower than that of the other classes. Therefore, water achieved the highest classification accuracy when using the six features derived from the coherency matrix and the covariance matrix. Furthermore, it can be seen in Table 4 that the producer accuracy (PA) and user accuracy (UA) of the two dryland crops (maize and cotton) both increase with evolution of the phenological period, irrespective of which classification algorithm is used. This demonstrates that higher accuracy of dryland crop classification can be achieved when using PolSAR imagery acquired in the late growing stage of the crops (i.e., maturation of maize and the wadding period of cotton). In comparison with the dryland crops, the PA and UA of the classification of buildings exhibit different trends of change with the evolution of crop phenological stage. The highest accuracy of classification of buildings was achieved at the jointing and budding period of maize and cotton. However, the PA and UA of the water classification remained stable and at a high level regardless of the phenological period. With regard to the classification algorithm, no significant difference was found between the OAs and Kappa coefficients derived using either the RF or the SVM algorithm. However, the running time of the RF algorithm was far shorter than that of the SVM algorithm. In addition, the PA and UA of the cotton classification using SVM were both zero, which is clearly not true. This indicates that the RF algorithm is more suitable than the SVM algorithm for performing land-cover classification using the PolSAR data adopted in this study.

### 4.2. Classification Performance with Multiple Features

In addition to the six backscattering intensity features derived from the Coherency Matrix and the Covariance Matrix, many other parameters extracted by polarization decomposition methods can also be used as features for land cover classification. The various accuracies and Kappa coefficients of the classification of the four land-cover types using 33 features extracted by the 2 polarization decomposition methods (i.e., 21 features from C–P decomposition and 12 features from scattering model decomposition) and the RF algorithm are listed in Table 5. Although the number of features increased from 6 to 33, the OAs and Kappa coefficients of the classification of the four land-cover types are not improved substantially in comparison with those achieved using the scattering intensity features. This demonstrates that satisfactory accuracy of dryland crop classification could not be achieved using only polarization decomposition parameters.

A feature set combining backscattering intensity and polarization decomposition parameters was used to improve the accuracy of the classification of the four land-cover types in this study. The accuracies and Kappa coefficients of the classification of the four land cover types using a combined dataset with 39 features (i.e., 6 intensity features, 21 C–P decomposition features, and 12 scattering model decomposition features) and the RF algorithm are listed in Table 6. Similar to the results shown in Table 5, the OAs and Kappa coefficients of the classification of the four land-cover types using the 39 features are not improved substantially, irrespective of the phenological period of the dryland crops in the study area. This indicates that the accuracy of the dryland crop classification could not be improved substantially when using only single PolSAR imagery capturing a certain phenological period, even though various features were used for the classification.

**Figure 4:**
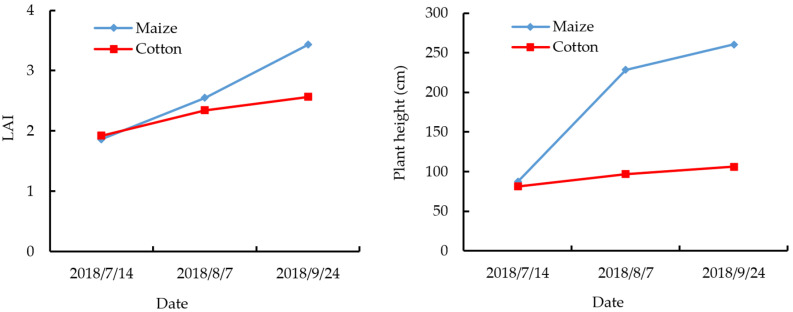
The leaf area index (LAI) and plant height (PH) of maize and cotton in three phenological stages.

In addition, as shown in Table 4, Table 5 and Table 6, the PAs and UAs of the dryland crop classification increase with evolution of the phenological stage, irrespective of whether backscattering intensity features only, polarization decomposition parameters only, or combinations of the two types of features are used. Specifically, when the maize and cotton crops were in the jointing and budding stages, the classification accuracy was lowest. Conversely, the maximum classification accuracy was achieved when both crops were in their early maturation stage. The reason is that maize and cotton have a very similar leaf area index (LAI) and PH in their jointing or budding stage (shown in Figure 4); consequently, the two crops had no obvious distinction in terms of geometric shape and structure. As the growing period developed, the maximum differences in LAI and PH between the two crop types occurred in their early maturation stage; hence maximum classification accuracy could be achieved. For buildings, the maximum and minimum classification accuracy occurred on 14 July 2018 and 7 August 2018, respectively. The reason is that the smaller LAI and PH of maize and cotton on the earlier date meant that their scattering type was mainly surface scattering, which differed from the scattering type associated with buildings. With evolution of the phenological period (i.e., by 7 August 2018), the PH of maize increased markedly but its LAI changed slowly (shown in Figure 4), which produced double scattering. This caused confusion between maize and buildings, which meant that the accuracy of the classification of buildings decreased.

### 4.3. Classification Performance Combining Multiple Features and Multitemporal Imagery

**Figure 5:**
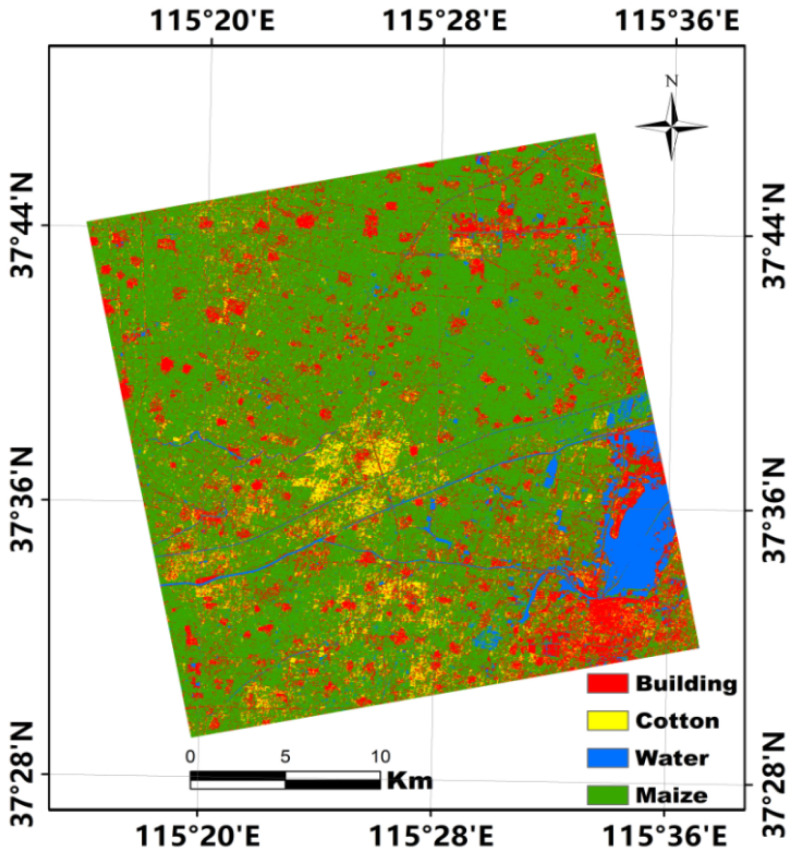
Classification map using 39 features with a single RADARSAT-2 image acquired on 24 September 2018.

**Figure 6:**
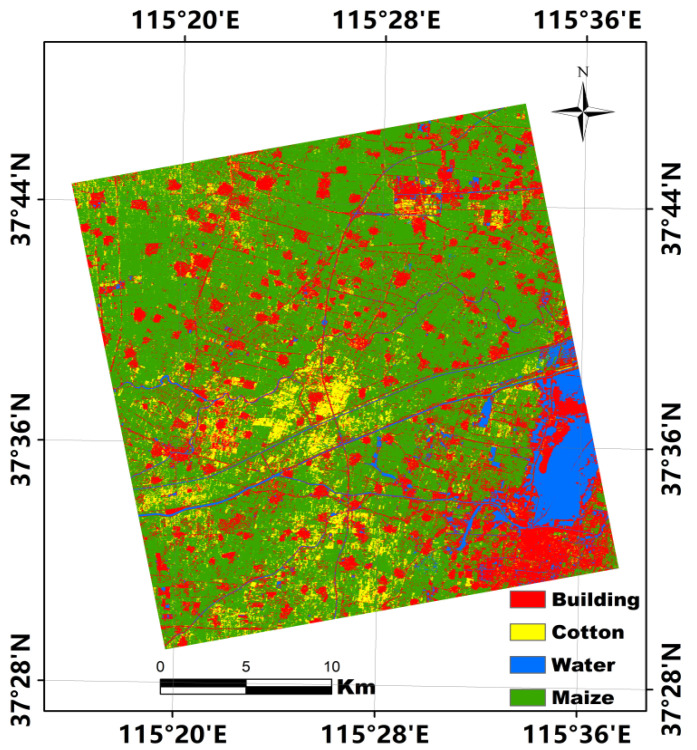
Classification map obtained using 117 features with 3 RADARSAT-2 images.

Multitemporal SAR imagery has often been used to improve crop classification accuracy because different crops show various distinctions in terms of their geometric shape and structure with evolution of the phenological period. As the accuracy of dryland crop classification was not improved substantially when using various features extracted from a single PolSAR images, we used three RADARSAT-2 PolSAR images that provided 117 features (each PolSAR image consisted of 39 backscattering intensity and polarization decomposition features) to improve the accuracy of dryland crop classification. A confusion matrix of the classification of the four land-cover types using 117 features and the RF algorithm is presented in Table 7 to assess quantitatively the classification results. In comparison with the classification results obtained using a single image, the OAs and Kappa coefficients of the classification of the four land-cover types using three temporal images are improved significantly. In particular, the PA and UA of the classification of maize are both >90%. For the classification of cotton, the PA is slightly lower than 90%; however, it is nearly 15% higher than that obtained using 39 features extracted from the single image acquired on 24 September 2018. This demonstrates that the accuracy of dryland crop classification can be improved substantially when using multitemporal PolSAR imagery. The classification map obtained using 39 features with a single RADARSAT-2 image (24 September 2018) is shown in Figure 5, and the classification map obtained using 117 features with three RADARSAT-2 images is shown in Figure 6. The former shows obvious manifestation of the “salt and pepper” phenomenon, especially in the classification results of buildings, whereas the latter presents clearer geometric shapes and boundaries with less evidence of the “salt and pepper” phenomenon.

In addition, we also employed a two-fold cross validation method to test the stability of four land-cover type classification accuracy using 117 features from the 3 full polarimetric RADARSAT-2 images. For the two-fold cross validation, the initial training samples presented in Table 2 were divided into two equal parts. One part of the samples were combined with the initial validation samples as the new training samples, and the other part was used as new validation samples in the first-fold cross validation; in turn, the new and initial validation samples were used as the training samples, and the first part of the initial training samples as the validation samples in the second-fold cross validation. The various accuracies and Kappa coefficient of four land cover types classification using 117 features and different training and validation samples are listed in Table 8. In terms of classification accuracy, the OA and Kappa coefficient of four typical ground objects are both high (OA > 90%, Kappa coefficient > 0.86), no matter which training samples were used. Moreover, the accuracies and Kappa coefficients presented in Table 8 are also quite similar to those of Table 7, although different training and validation samples were used in the two tables. This demonstrates that a stable accuracy can be achieved when 117 features from the 3 full polarization RADARSAT-2 images were used to classify the four typical ground objects in this study area.

### 4.4. Assessment of Importance of All Features Extracted from Three RADARSAT-2 Images

**Figure 7:**
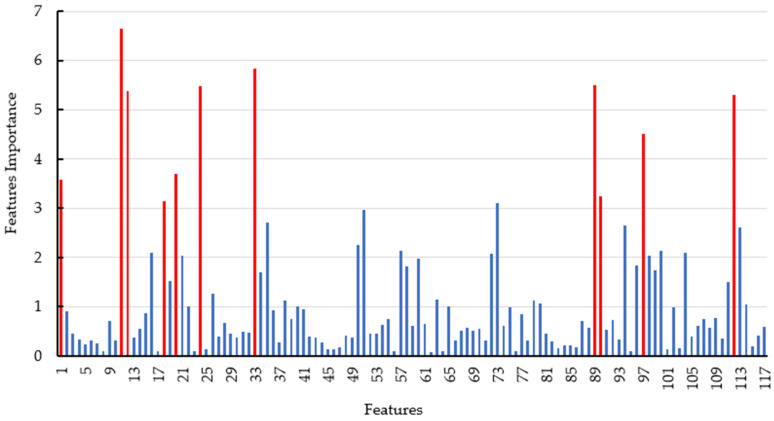
Bar graph of average values of the importance of 117 features extracted from 3 RADARSAT-2 images for dryland crop classification. Features 1–39, 40–78, and 79–117 were extracted from images acquired on 14 July, 7 August, and 24 September 2018, respectively. The names of features 1–39 are listed in Table 3. Features 40–78 and 79–117 have the same names as features 1–39. Red bars denote features ranking in the top 11 in terms of importance.

**Figure 8:**
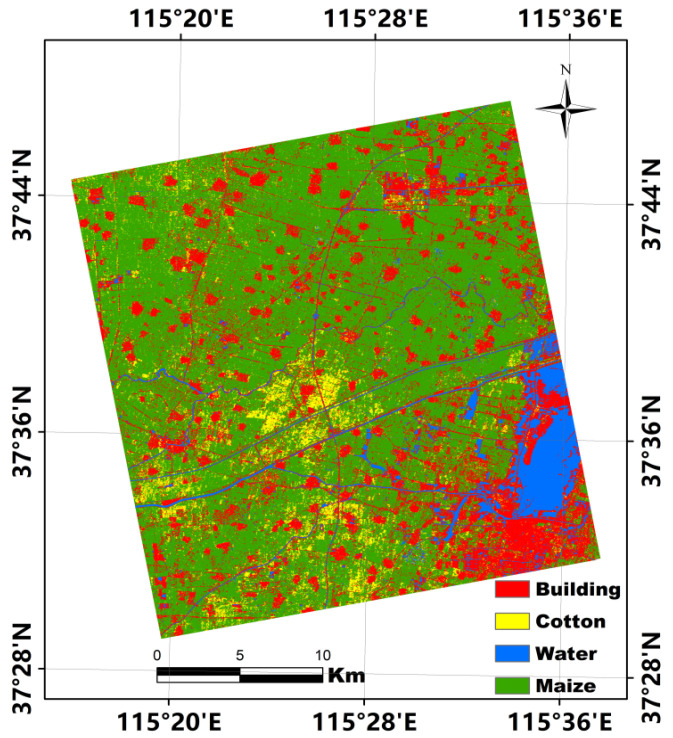
Classification map obtained using 11 features with 2 RADARSAT-2 images.

Although the accuracy of the dryland crop classification achieved using 117 features from the 3 full polarization RADARSAT-2 images was satisfactory, the high number of features used for the classification generated a heavy workload and the acquisition cost of the RADARSAT-2 imagery was high. Therefore, assessment of the importance of the features used is necessary for optimization of both the number and types of the features and the number and acquisition dates of the RADARSAT-2 imagery. In this study, the importance of all 117 features was evaluated quantitatively using the RF algorithm. The relative importance of each of the 117 features extracted from the 3 RADARSAT-2 images for dryland crop classification is illustrated in the bar graph in Figure 7. The 11 features ranking highest in terms of importance are SE (No. 11), T11 (No. 33), SE (No. 89), Lambda (No. 33), SE_I_ (No. 12), T22 (No. 112), P2 (No. 92), P3 (No. 20), Alpha (No. 1), SE_I_ (No. 90), and P1 (No. 18). Interestingly, the importance of each of most of the remaining features is comparatively much lower. Specifically, the top 11 features were derived from 2 RADARSAT-2 images. The features of No. 1, No. 11, No. 12, No. 18, No. 20, No. 24, and No. 33 were from the RADARSAT-2 image acquired on 14 July 2018, and the remaining features were from the image acquired on 24 September 2018. In addition, all 11 features were extracted using the C–P polarization decomposition approach and the coherency matrix. We then used the top 11 features to distinguish the dryland crops and other land cover types in the study area because their contributions to the classification far exceeded those of the remaining features. The confusion matrix of the classification of the four land-cover types using those 11 features and the RF algorithm is presented in Table 9. In comparison with the results obtained using 117 features (Table 7 and Table 8), accuracy decreased slightly in the classification using only 11 features. However, the OA of the classification result is >90% and the Kappa coefficient is >0.8. The classification map obtained using the top 11 features and the RF algorithm is shown in Figure 8. Similar to the classification map obtained using 117 features (Figure 6), the map obtained using 11 features also has clear outlines and boundaries with less evidence of the “salt and pepper” phenomenon. This analysis demonstrates that a satisfactory level of accuracy could be achieved in classification of the 4 land cover types using only 11 features extracted from 2 RADARSAT-2 PolSAR images.

In order to verify the stability of four land-cover types’ classification accuracy using 11 optimized features from 2 full polarization RADARSAT-2 images, we still used the same rotation samples presented in Table 8 to implement a two-fold cross validation. The various accuracies and Kappa coefficient of four land cover types classification using 11 features and different training and validation samples are listed in Table 10. Similar to the results in Table 8, a high accuracy was achieved regardless of samples rotation, although the number of features for classification has been reduced from 117 to 11. Furthermore, the accuracies and Kappa coefficients presented in Table 10 are also very close to those of Table 9, even though different training and validation samples were used to classify four typical ground objects in the study area. This demonstrates that a stale and satisfied accuracy can be achieved when using 11 optimized features from 2 full polarization RADARSAT-2 images.

## 5. Discussion

Hebei plain is an important area of grain and cotton production in China. Maize and cotton are two predominant dryland crops cultivated in Hebei plain, and their production provides an important contribution to the income of farmers and ensures China’s food security. Timely and accurate classification of maize and cotton plays a key role in forecasting dryland crops yields and enhancing agricultural management in Hebei plain. However, owing to the cloudy/rainy weather that occurs frequently during the growing period of the two dryland crops, remotely sensed optical imagery suitable for dryland crop classification is often unavailable. Although satellite-based SAR imagery with its all-weather observation capability has been used to overcome this shortcoming, the accuracy of dryland crop classification using single-date satellite-based SAR data is generally not high. In this study, we chose Jizhou county which can represent the cropping characteristic of Hebei plain as the study area, and analyzed the influence of various features and acquisition dates of polarimetric RADARSAT-2 images on the accuracy of the two dryland crops’ classification; furthermore, the number of features and PolSAR images was optimized by the quantitative assessment of feature importance. Specifically, satisfactory accuracy in the classification of dryland crops was achieved using 11 optimized features (extracted using the C–P polarization decomposition approach and the Coherency Matrix) and 2 RADARSAT-2 images (acquisition dates corresponding to the middle and late growing stages of the dryland crops). Finally, a stable accuracy can be achieved using the dryland crop classification method proposed in the study, based on a two-fold cross validation. In this way, this study illustrates key features and phenological stage suitable for dryland crop classification by satellite-based PolSAR image, and improves the accuracy of dryland crop classification significantly only using 11 features from 2 full polarization RADARSAT-2 images. Furthermore, this classification method combining multitype features and multitemporal PolSAR images also provides a reference for timely and accurate classification of dryland crop in Hebei plain, China.

We compared quantitatively the accuracy of dryland crop classification using full polarimetric RADARSAT-2 imagery with different acquisition dates. We demonstrated that higher classification accuracy could be achieved using multitemporal PolSAR imagery in comparison with that of single-date imagery, which is consistent with the findings of previous studies [20,24,28,46]. Based on observational results of LAI and PH of the studied dryland crops in three phenological stages corresponding to the different acquisition dates of the RADARSAT-2 images, we also analyzed the effect of acquisition date of PolSAR imagery on the accuracy of dryland crop classification. We demonstrated that the highest classification accuracy could be achieved using imagery that corresponded to the early maturation period of the dryland crops. This finding differs from previous studies that considered only the relative classification accuracies achieved on specific dates of imagery acquisition. Furthermore, in previous studies, the time of imagery acquisition was not associated with the phenological stage of the crops, and the differences in classification accuracy were not analyzed or explained in relation to the different dates of acquisition of the SAR imagery. Regarding the extraction and selection of features, we used various approaches and 3 full polarimetric RADARSAT-2 images to extract 117 features for dryland crop classification. Based on assessment of the relative importance of all the features, it was demonstrated that the Shannon entropy extracted using the C–P polarization decomposition method made an important contribution to the accuracy of dryland crop mapping. This finding is also in accord with the results of previous research [9]. The backscattering coefficient in different polarization modes and the polarization decomposition parameters extracted using the C–P, F–D, and Yamaguchi decomposition methods have been used widely for land-cover and land-use classification. In addition to these features, we also used other backscattering intensity features, e.g., the main diagonal elements in the Coherency Matrix, and the polarization parameters extracted using the MCSM decomposition approach to enhance the accuracy of dryland crop classification. This novel combination of various features is markedly different from that of previous studies [9,20,29,35]. For optimization of the process, we used the RF algorithm to identify the most important features and the critical dates of imagery acquisition, the use of which was shown to both improve significantly the efficiency of crop classification and reduce the workload and costs associated with crop mapping. This represents the innovation of this study.

Although the OA of dryland crop classification using the proposed method exceeded 90%, the PA of the classification of cotton remained unsatisfactory. Moreover, the results of the classification of cotton were found to often vary greatly with different features and different dates of acquisition of SAR imagery. As shown in Figure 6, maize is the dominant crop in the study area and it is often cultivated in large plots. In contrast, cotton is often cultivated in small-sized scattered plots that might be overlooked in the crop classification process, which could lead to large omission errors. Therefore, greater attention should be paid to minor crops (e.g., cotton) cultivated in small-sized plots in further study. In addition, we used only three images with different acquisition dates to analyze the classification performance of the full polarimetric RADARSAT-2 data. However, three dates cannot cover the entire phenological period of dryland crops. Therefore, additional imagery corresponding to each growth stage of the crops will be required in future research to determine the optimal imagery acquisition times and features required for accurate and high-efficiency dryland crop classification.

## 6. Conclusions

In this study, an in-depth investigation was conducted into the accuracy of the classification of two typical dryland crops (maize and cotton) in Jizhou county, Hebei plain, China using satellite-based PolSAR imagery. Three quad-polarimetric RADARSAT-2 images, 117 features extracted using different approaches and images, and 2 algorithms were used to classify the two dryland crops and other typical ground objects in the study area. The accuracies of dryland crop classification using different features, images, and algorithms were compared quantitatively, and the relative importance of each of the features used in the classification process was assessed. Finally, the optimal features, algorithm, and phenological period for dryland crop classification using RADARSAT-2 PolSAR imagery were proposed. The main conclusions of this study are as follows.

First, the accuracy of dryland crop classification increased with the evolution of the phenological period. For the three growing periods investigated in this study, maximum accuracy was achieved using quad-polarimetric RADARSAT-2 imagery acquired in the early maturation stage of the dryland crops. In comparison with the SVM algorithm, the RF algorithm was found most suitable for dryland crop classification using satellite-based PolSAR imagery.

Second, dryland crop classification accuracy was not improved substantially when using only backscattering intensity or polarization decomposition features extracted from a single RADARSAT-2 image. However, satisfactory accuracy could be achieved using a combination of multitype features and multitemporal RADARSAT-2 imagery.

Finally, based on assessment of the relative importance of all the features, 11 features (extracted using the C–P decomposition method and Coherency Matrix) and 2 phenological stages (the jointing or budding stage of maize and cotton and their maturation stage) were found to make important contributions to the accuracy of the classification of dryland crops. A high and reliable accuracy of dryland crop classification can be achieved only using the 11 features from 2 full polarization RADARSAT-2 images, based on a two-fold cross validation.

## Figures and Tables

**Table 1:** Description of the acquired RADARSAT-2 polarimetric synthetic aperture radar (PolSAR) data.

Date	Mode	Orbit	Look Direction	Phenological Stage	
				Maize	Cotton
20180714	FQ 7	Ascending	Right	Jointing	Budding
20180807	FQ 7	Ascending	Right	Heading	Blossoming and boll-forming
20180924	FQ 7	Ascending	Right	Maturation	Wadding

**Table 2:** Field data collected and the numbers of pixels used for classification training and testing.

Land Cover	Training Samples		Testing Samples	
	Number of Fields	Number of Pixels	Number of Fields	Number of Pixels
Maize	67	45,691	34	24,438
Cotton	76	9405	39	4859
Water	67	16,324	33	4758
Buildings	68	29,343	34	12,703

**Table 3:** Overview of the 39 features extracted from the RADARSAT-2 PolSAR data.

Number	Features	Abbreviation
1	Average scattering alpha angle	Alpha
2	Anisotropy	Anisotropy
3	Target Randomness	P_R_
4	Average scattering beta angle	Beta
5	Main diagonal element 1 of [*C*_3_]	*C*_11_
6	Main diagonal element 2 of [*C*_3_]	*C*_22_
7	Main diagonal element 3 of [*C*_3_]	*C*_33_
8	Average scattering delta angle	Delta
9	Double Bounce Eigenvalue Relative Difference	DERD
10	Entropy	Entropy
11	Shannon Entropy	SE
12	Intensity component of SE	SE_I_
13	Polarization component of SE	SE_P_
14	Freeman-odd	F-odd
15	Freeman-dbl	F-dbl
16	Freeman-vol	F-vol
17	Average scattering gamma angle	Gamma
18	Probability 1	P1
19	Probability 2	P2
20	Probability 3	P3
21	The first eigenvector	L1
22	The second eigenvector	L2
23	The third eigenvector	L3
24	The Average eigenvector	Lambda
25	Multiple-Component Scattering Model (MCSM)-odd	M-odd
26	MCSM-dbl	M-dbl
27	MCSM-vol	M-vol
28	MCSM-hlx	M-hlx
29	MCSM-wire	M-wire
30	Pedestal Height	PH
31	Radar Vegetation Index	RVI
32	Single Bounce Eigenvalue Relative Difference	SERD
33	Main diagonal element 1 of [*T*_3_]	*T*_11_
34	Main diagonal element 2 of [*T*_3_]	*T*_22_
35	Main diagonal element 3 of [*T*_3_]	*T*_33_
36	Yamaguchi-odd	Y-odd
37	Yamaguchi-dbl	Y-dbl
38	Yamaguchi-vol	Y-vol
39	Yamaguchi-hlx	Y-hlx

**Table 4:** Accuracies and Kappa coefficients of the classification of four land-cover types using six features and two algorithms. (PA: producer accuracy, UA: user accuracy, OA: overall accuracy, Kappa: Kappa coefficient, Time: running time for classification).

Date	PA (%)				UA (%)				OA (%)	Kappa	Time (min)
	Maize	Cotton	Water	Building	Maize	Cotton	Water	Building			
	**RF**										
20180714	87.64	9.03	89.40	77.25	75.20	35.94	98.53	78.40	77.31	0.619	58
20180807	91.10	55.65	93.01	54.13	76.82	73.34	98.51	72.25	78.10	0.637	64
20180924	91.97	60.83	93.11	60.36	80.13	78.43	98.91	73.39	80.89	0.669	61
	**SVM**										
20180714	92.42	0.00	93.87	78.61	75.21	0.00	99.86	81.88	79.29	0.644	1098
20180807	93.56	57.79	92.40	55.63	77.29	79.10	99.70	78.42	79.80	0.659	1086
20180924	95.72	65.82	92.45	58.30	79.67	88.63	98.83	79.32	81.20	0.687	1063

**Table 5:** Accuracies and Kappa coefficients of the classification of 4 land-cover types using 33 polarization decomposition features and the random forest algorithm.

Date	PA (%)				UA (%)				OA (%)	Kappa
	Maize	Cotton	Water	Building	Maize	Cotton	Water	Building		
20180714	89.06	9.30	93.51	81.41	76.76	36.22	98.27	82.08	79.24	0.652
20180807	91.61	59.77	93.62	58.37	77.95	75.17	98.32	75.04	79.86	0.667
20180924	92.28	63.50	93.18	64.58	81.27	81.95	98.12	76.01	81.42	0.696

**Table 6:** Accuracies and Kappa coefficients of the classification of 4 land-cover types using 39 features and the random forest algorithm.

Date	PA (%)				UA (%)				OA (%)	Kappa
	Maize	Cotton	Water	Building	Maize	Cotton	Water	Building		
20180714	89.37	9.61	93.81	81.46	76.79	36.50	98.57	82.32	79.35	0.653
20180807	92.01	60.77	93.62	58.83	78.08	76.98	98.52	75.94	79.98	0.669
20180924	98.63	63.67	93.04	64.61	81.38	82.35	98.15	76.49	81.52	0.697

**Table 7:** Confusion matrix of the 4 land-cover types classified using 117 features and the random forest algorithm.

Class	Ground Truth					
	Maize	Cotton	Water	Building	Total	UA (%)
Maize	23,524	804	68	896	25,292	93.01
Cotton	124	3721	0	188	4033	92.26
Water	2	0	4510	15	4527	99.62
Building	768	287	163	11578	12,796	90.48
Total	23,524	4812	4761	12677	46,648	
PA (%)	96.34	77.33	95.13	91.33		
OA (%)	92.89					
Kappa	0.886					

**Table 8:** Accuracies and Kappa coefficients of the classification of four land-cover types using 117 features and different training and validation samples. (TS_1_: the initial training samples presented in Table 2, 1/2TS_1_ denotes a half of the initial training samples, TS_r_: the other half of the initial training samples, VS_1_: the initial validation samples presented in Table 2).

Categories	Maize	Cotton	Water	Building	OA (%)	Kappa	Training Samples	Validation Samples
PA (%)	96.19	79.36	95.85	87.36	91.88	0.870	1/2TS_1_ + VS_1_	TS_r_
UA (%)	90.98	88.66	96.24	93.21				
PA (%)	97.16	77.08	95.03	93.01	93.51	0.899	TS_r_ + VS_1_	1/2TS_1_
UA (%)	92.70	89.29	99.91	94.02				

**Table 9:** Confusion matrix of the 4 land cover types classified using 11 features and the random forest algorithm.

Class	Ground Truth					
	Maize	Cotton	Water	Building	Total	UA (%)
Maize	23,226	1255	74	1185	25,740	85.71
Cotton	284	3219	0	250	3756	85.70
Water	4	1	4470	82	4527	98.09
Building	901	344	0	11,160	12,796	88.71
Total	24,418	4819	4720	12,677	46,648	
PA (%)	95.12	66.80	94.70	88.03		
OA (%)	90.22					
Kappa	0.842					

**Table 10:** Accuracies and Kappa coefficients of the classification of four land-cover types using 11 features and different training and validation samples. (TS_1_, TS_r_ and VS_1_ denotes the same meaning as in Table 8).

Categories	Maize	Cotton	Water	Building	OA (%)	Kappa	Training Samples	Validation Samples
PA (%)	92.86	64.01	96.06	86.44	89.81	0.826	1/2TS_1_ + VS_1_	TS_r_
UA (%)	89.82	81.68	94.34	88.45				
PA (%)	95.85	65.67	94.73	91.55	91.50	0.865	TS_r_ + VS_1_	1/2TS_1_
UA (%)	90.50	82.44	99.60	92.94				

## Data Availability

Data sharing not applicable.
